# THE COMPLEXITY OF DUAL PATHOLOGY: REGARDING A CASE REPORT OF SEIZURES

**DOI:** 10.1192/j.eurpsy.2023.1603

**Published:** 2023-07-19

**Authors:** M. F. De Andrade, M. Magalhães, L. Gil, V. Viveiros, C. Moreira

**Affiliations:** Centro Hospitalar Psiquiátrico de Lisboa, Lisbon, Portugal

## Abstract

**Introduction:**

Wernicke’s encephalopathy (WE) is a potentially reversible neuropsychiatric emergency caused by thiamine deficiency, whose classical triad consists of acute onset of confusion, gait ataxia, and oculomotor dysfunction. The diagnosis is missed in 75-80% of cases and approximately 80% of untreated patients develop Korsakoff Syndrome, which is characterized by memory impairment associated with confabulation. Early recognition of nutritional deficiency or any portion of the triad is critical and should prompt treatment, since WE is readily reversible if treated with adequate doses of parenteral thiamine.

**Objectives:**

Starting from a case report of suspected WE, we pretend to discuss the differential diagnosis of seizures in dual pathology.

**Methods:**

Non-systematic review of the literature was performed in PubMed database using the keywords “Wernicke’s Encephalopathy”, “Seizures”, “Alcohol” and “Benzodiazepines”. The articles were selected according to their relevance. A patient´s clinical record was reviewed and presented.

**Results:**

We present a case of a 44-year-old Ukrainian man with suspected background of chronic alcohol abuse and psychiatric history of schizoaffective disorder, who presented with acute onset of confusion, psychomotor agitation, gait ataxia and nystagmus. Anamnesis was hampered by the language barrier and absence of past medical history and patient’s alcoholic habits remained unclear. After suspicion of WE it was introduced thiamine and diazepam, with significant improvement. After discontinuation of diazepam, the patient presented with several episodes of tonic-clonic seizures. He was medicated for seizures with clinical stabilization. At time of discharge the diagnostic discussion prevailed.

Seizures are a common presentation of various conditions associated with alcohol use, whose differential diagnosis is difficult, especially in patients with dubious alcohol consumption. Alcohol abuse is a major precipitant of status epilepticus as seizure threshold is raised by alcohol drinking. Seizures may also occur during alcohol withdrawal, for which treatment with benzodiazepines is recommended, however carefully, since both abrupt cessation and high-dose use are critical for the appearance of seizures. Although very rare, WE may also present with seizures, whereby overdiagnosis and overtreatment are preferred to prevent persistent neurocognitive impairments.

**Conclusions:**

This case illustrates the complexity of neuropsychiatric diagnoses in dual pathology. It requires a longitudinal assessment for a better understanding of clinical conditions and establishment of the best therapeutic approach.

**Disclosure of Interest:**

None Declared

